# P-1772. Diagnostic Utility of Fluorescent Antibody Microscopy (FAM) for Giardiasis and Cryptosporidiosis

**DOI:** 10.1093/ofid/ofaf695.1942

**Published:** 2026-01-11

**Authors:** Akira Kawashima, Yusuke Oshiro, Megumi Akashi, Yasuaki Yanagawa, Rieko Shimogawara, Masami Kurokawa, Naokatsu Ando, Haruka Uemura, Takahiro Aoki, Kei Yamamoto, Junichi Akiyama, Daisuke Mizushima, Kenji Yagita, Hiroyuki Gatanaga, Koji Watanabe

**Affiliations:** National Center for Global Health and Medicine, Japan Institute for Health Security, Shinjuku-ku, Tokyo, Japan; National Center for Global Health and Medicine, Japan Institute for Health Security, Shinjuku-ku, Tokyo, Japan; National Center for Global Health and Medicine, Japan Institute for Health Security, Shinjuku-ku, Tokyo, Japan; National Center for Global Health and Medicine, Japan Institute for Health Security, Shinjuku-ku, Tokyo, Japan; National Institute of Infectious Diseases, Japan Institute for Health Security, Shinjuku-ku, Tokyo, Japan; National Center for Global Health and Medicine, Japan Institute for Health Security, Shinjuku-ku, Tokyo, Japan; National Center for Global Health and Medicine, Japan Institute for Health Security, Shinjuku-ku, Tokyo, Japan; National Center for Global Health and Medicine, Japan Institute for Health Security, Shinjuku-ku, Tokyo, Japan; National Center for Global Health and Medicine, Japan Institute for Health Security, Shinjuku-ku, Tokyo, Japan; National Center for Global Health and Medicine, Japan Institute for Health Security, Shinjuku-ku, Tokyo, Japan; National Center for Global Health and Medicine, Japan Institute for Health Security, Shinjuku-ku, Tokyo, Japan; National Center for Global Health and Medicine, Japan Institute for Health Security, Shinjuku-ku, Tokyo, Japan; National Institute of Infectious Diseases, Japan Institute for Health Security, Shinjuku-ku, Tokyo, Japan; National Center for Global Health and Medicine, Japan Institute for Health Security, Shinjuku-ku, Tokyo, Japan; Tokai University School of Medicine, Isehara-shi, Tokyo, Japan

## Abstract

**Background:**

Giardiasis and Cryptosporidiosis are common diarrheal diseases that are often underdiagnosed due to non-specific symptoms and diagnostic limitations. While multiplex-PCR and rapid antigen tests (rapid-IC) offer high accuracy, their cost and restricted pathogen coverage, especially for parasites, limit their use. This study evaluates newly developed diagnostic method for these protozoa, named fluorescent antibody microscopy (FAM).

**TABLE 1:**
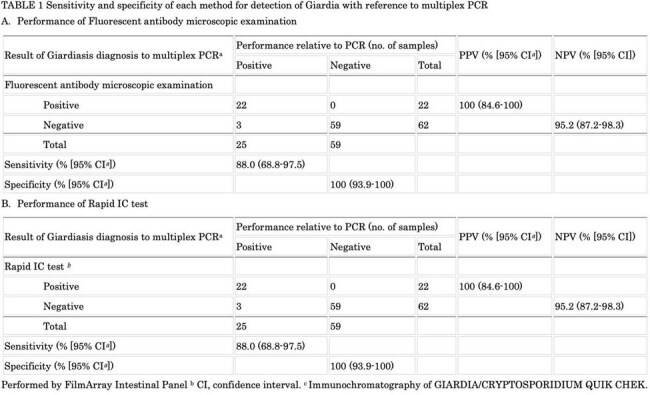
Sensitivity and specificity of each method for detection of Giardia with reference to multiplex PCR

**TABLE 2:**
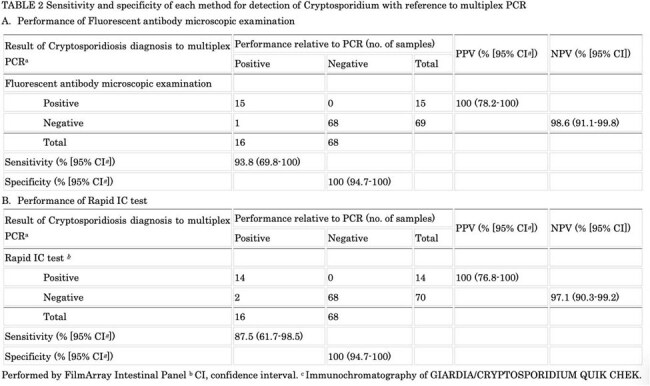
Sensitivity and specificity of each method for detection of Cryptosporidium with reference to multiplex PCR

**Methods:**

For performing FAM, stool samples are mixed with DyLight 488-conjugated antibodies against *G. duodenalis* cysts and *Cryptosporidium*oocysts, thereafter, the samples are examined by fluorescence microscopy to detect these protozoa. All samples showing positive for FAM, and unbiasedly selected those with FAM negative result were subjected to multiplex-PCR, and rapid-IC. Diagnostic accuracies of FAM and rapid-IC were assessed by the result of multiplex-PCR as a reference standard. Cases with discordant results among these tests were examined by conventional PCR with sequencing to confirm infection.

**Results:**

FAM was examined for 694 stool samples during study period, which identified 35 “FAM positive samples”. Also, “FAM negative samples” were selected from all cases with negative results at any one month (49 samples). In total, a subset of 84 samples underwent further analysis. For Giardiasis, FAM showed 88.0% sensitivity and 100% specificity, which was exactly the same results with rapid-IC, including 3 samples showing false negative by both tests. (Table 1) For cryptosporidiosis, FAM had 93.8% sensitivity and 100% specificity, which represents comparable diagnostic accuracy with rapid-IC. (Table 2) Interestingly, conventional PCR for *G. duodenalis* oppositely showed negative results for 3 samples with multiplex-PCR positive, and FAM/rapid-IC negative results, which raised 2 possibilities; false negative results of FAM/rapid-IC due to extremely low pathogen burden, or false positive result of multiplex-PCR.

**Conclusion:**

FAM, a newly developed low-cost morphological stool examination for *Giardia* and *Cryptosporidium*, showed comparably high diagnostic accuracy with rapid-IC. It could be a cost-effective option for routine stool testing.

**Disclosures:**

All Authors: No reported disclosures

